# Metformin reduces the risk of developing influenza A virus related cardiovascular disease

**DOI:** 10.1016/j.heliyon.2023.e20284

**Published:** 2023-09-21

**Authors:** Han Sol Lee, Ji Yun Noh, Joon Young Song, Hee Jin Cheong, Woo Joo Kim

**Affiliations:** a Asia Pacific Influenza Institute, Guro Hospital, Korea University College of Medicine, Seoul, Republic of Korea; b Division of Infectious Diseases, Department of Internal Medicine, Guro Hospital, Korea University College of Medicine, Seoul, Republic of Korea

**Keywords:** Influenza A virus, Metformin, Cardiovascular diseases, MCP-1, IP-10

## Abstract

This study investigated the drug repositioning potential of metformin for cardiovascular risk due to influenza A virus infection. Statistical analysis was performed to analyze factors related to the risk of death after IAV infection in diabetic patients. Through *in vitro* and *in vivo* experiments, the effect of metformin on influenza A virus infection in non-diabetic conditions was analyzed. In logistic regression analysis, influenza vaccination (OR = 0.378, p-value = 0.007, 0.186 < 95% C·I < 0.768) and metformin treatment (OR = 0.380, p-value = 0.016, 0.173 < 95% C·I < 0.835) were associated with a decreased influenza-related mortality in diabetic patients. *In vitro* and *in vivo* studies showed that viral replication and influenza A virus-induced cytokine expression were inhibited by metformin. In particular, MCP-1 and IP-10, cytokines related to cell infiltration and cardiovascular disease development, were significantly reduced by metformin under influenza A virus infection condition. As a result, the acute exacerbation of atherosclerosis caused by influenza A virus in mouse aorta was inhibited by metformin. In addition, we found that regulation of AKT/MAPK signaling plays an important role in the mechanism of metformin. In conclusion, we demonstrated the potential and mechanism of metformin as a treatment for acute exacerbation of atherosclerosis caused by influenza A virus infection.

## Highlights

• Metformin has multiple mechanisms and regulates various cellular responses.
• Metformin is associated with a decreased influenza-related mortality in patients with diabetes.
• MCP-1 and IP-10 were reduced by metformin in influenza A virus infected mice.
• Metformin reduced exacerbation of atherosclerosis caused by influenza A virus.

## 1 Introduction

Coronary artery disease (CAD) is the most common form of heart disease and a leading cause of death worldwide [1,2]. The timely management of risk factors is important to reduce morbidity and mortality in CAD patients. Various factors are considered as risk factors for CAD, such as tobacco, obesity, high blood pressure, high cholesterol, diabetes mellitus (DM), and inflammation caused by pathogenic infection [[3], [4], [5], [6]]. Metformin, a first line treatment of type II diabetes, increases insulin sensitivity in the liver and peripheral tissues, reduces gluconeogenesis, and decreases inflammatory cytokines [[7], [8], [9], [10], [11]]. Treating DM patients with metformin improved cardiovascular risk mediated by weight loss, improved insulin resistance, decreased metabolic syndrome, and decreased cholesterol [[12], [13], [14]]. Metformin is also being studied for its potential as a therapeutic agent for cancer or inflammatory diseases [15,16]. In a previous study, we identified increased serum matrix metalloproteinase-13 (MMP-13) in influenza A virus (IAV)-infected patients and reported that increased MMP-13 caused by IAV infection induced acute cardiovascular events mediated by atherosclerotic plaque rupture [17]. In that study, we observed that the IAV-induced MMP-13 expression was inhibited by metformin. We hypothesized that metformin reduces the exacerbation of underlying diseases caused by IAV infection. Based on previous results, we investigated the cardioprotective, immunomodulatory and antiviral effects of metformin. The aim of this study was to determine whether metformin can effectively ameliorate acute cardiovascular events caused by IAV and to elucidate the underlying mechanism.

## 2 Results

### 2.1 Statistical analysis of influenza-related death

Statistical analysis was performed to analyze factors related to the risk of death after IAV infection in DM patients. Among the 654 subjects, there was no significant differences in age (IAV-related death group = 73.3 ± 11.4; Alive group = 71.8 ± 11.4), blood glucose concentration (IAV-related death group = 204.7 ± 120.6; Alive group = 188.0 ± 99.8), and number of underlying diseases (IAV-related death group = 2.4 ± 0.8; Alive group = 2.3 ± 0.9) between the IAV-related death group (n = 44) and the alive group (n = 610). As a result of correlation analysis between IAV-related death and each variable, it was confirmed that influenza vaccination and metformin treatment had a significant correlation with IAV-related death (Table 1). As a results of logistic regression analysis, we confirmed that not only Flu vaccination but also using metformin significantly reduce the risk of death associated with IAV in DM patients. In particular, influenza vaccination was associated with a decreased risk of IAV-related death by 63% (p-value = 0.007, 0.186 < 95% C·I < 0.768), and metformin administration was associated with a decreased risk of IAV-related death by 62% (p-value = 0.016, 0.173 < 95% C·I < 0.835) (Table 1). In conclusion, metformin is associated with a decreased influenza-related mortality risk. Thus, we focused on the immunomodulatory and antiviral effects of metformin, and conducted *in vitro* and *in vivo* studies to confirm the effect of metformin on Influenza infection.

**Table 1:** Baseline characteristics of study participants and statistical analysis results.

Variable	Number	Groups		Correlation analysis				logistic regression analysis						
		IAV-related Death (n = 44)	Alive (n = 610)	p-value	OR	95% CI		B	S.E.	Wald	p-value	OR	95% CI	
Age	71.9 ± 11.4	73.3 ± 11.4	71.8 ± 11.4	0.406	0.988	0.961	1.016	−0.024	0.017	2.064	0.151	0.976	0.944	1.009
Sex				0.148	0.630	0.336	1.179	−0.767	0.359	4.556	0.033*	0.464	0.230	0.939
Female	322	17	305											
Male	332	27	305											
History of Diseases														
Cadiovascular/Cerebrovascular disease	362	27	335	0.407	1.304	0.696	2.442	0.312	0.356	0.766	0.381	1.366	0.680	2.743
Neuromuscular disease	31	1	30	0.437	0.450	0.060	3.377	−0.889	1.072	0.688	0.407	0.411	0.050	3.360
Respiratory disease	168	11	157	0.914	0.962	0.475	1.949	−0.112	0.393	0.081	0.776	0.894	0.414	1.933
Liver/Kidney disease	167	15	152	0.181	1.559	0.814	2.985	0.446	0.380	1.375	0.241	1.561	0.741	3.288
Cancer	96	8	88	0.498	1.318	0.593	2.930	0.312	0.433	0.519	0.471	1.366	0.584	3.194
Autoimmune disease	14	1	13	0.950	1.068	0.136	8.357	0.365	1.108	0.108	0.742	1.440	0.164	12.624
Smoking status				0.318	0.670	0.305	1.472	−0.761	0.440	2.993	0.084	0.467	0.197	1.106
Current smoker	160	8	152											
Never or former smoker	494	36	458											
Number of Chronic diseases	2.3 ± 0.9	2.4 ± 0.8	2.3 ± 0.9	0.270	0.824	0.584	1.162							
Glucose	189.2 ± 101.3	204.7 ± 120.6	188.0 ± 99.8	0.294	0.999	0.996	1.001	−0.002	0.001	1.766	0.184	0.998	0.995	1.001
Flu Vaccination*	438	23	415	0.034*	0.515	0.278	0.952	−0.972	0.362	7.228	0.007*	0.378	0.186	0.768
S.Pneumoniae vaccination	269	20	249	0.547	1.208	0.653	2.235	0.343	0.367	0.872	0.351	1.409	0.686	2.896
Type of influenza Virus														
Influenza A virus	540	34	506	0.340	1.431	0.686	2.987	0.496	1.179	0.177	0.674	1.641	0.163	16.538
Influenza B virus	123	11	112	0.279	1.482	0.727	3.022	0.792	1.141	0.482	0.488	2.209	0.236	20.691
Anti-viral drug	609	40	569	0.550	0.721	0.246	2.112	−0.366	0.602	0.371	0.543	0.693	0.213	2.254
Anti-biotics	571	44	527	0.997	134878355	0.000	.							
Hyperlipidemia medications														
IStatin	238	14	224	0.515	0.804	0.418	1.549	−0.191	0.365	0.275	0.600	0.826	0.404	1.688
INon-statin	15	1	14	0.992	0.990	0.127	7.708	0.270	1.087	0.062	0.804	1.309	0.156	11.016
Antidiabetic drugs														
IMetformin*	314	14	300	0.029*	0.482	0.251	0.927	−0.966	0.401	5.811	0.016*	0.380	0.173	0.835
INon-metformin	115	6	109	0.062	0.385	0.141	1.048	−0.907	0.529	2.943	0.086	0.404	0.143	1.138
IInsulin	158	11	147	0.893	1.050	0.518	2.129	−0.531	0.443	1.438	0.230	0.588	0.247	1.401

OR, odd ratio; CI, confidence interval.

*indicated statistically significant (p-value <0.05).

### 2.2 Antiviral effect of metformin in influenza A virus infected cells

**Fig. 1:**
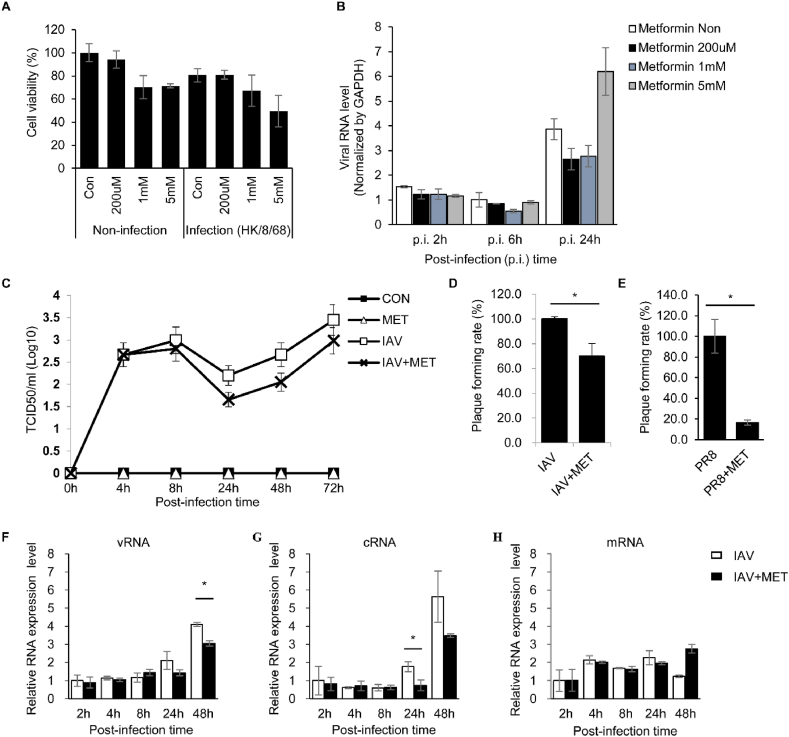
**The replication of Influenza A virus was suppressed by metformin in the A549 cells infected with influenza A virus.** A549 cells were infected with mock or influenza A virus (HK/8/68) at a multiplicity of infection (MOI) of 1 or 0.1. One hour after infection, the media was replaced and metformin (0–5 mM) was treated. (**A)** Cell viability was measured using WST assay after 48 h of incubation. (**B)** Total RNA was isolated from influenza A virus-infected cells at each time point. The M gene of influenza A virus and GAPDH were measured using quantitative real-time PCR. Viral RNA levels of influenza A virus in cells were normalized by GAPDH. (**C)** Virus concentration in the cell culture supernatant was measured by TCID_50_ assay for each time point. (**D** and **E)** plaque assays were performed using supernatants collected 72 h after influenza A virus infection. After infection with (**D**) influenza A virus H3N2 strain (IAV) or (**E**) H1N1 strain (PR8), the plaque formation unit of supernatant isolated from PBS-treated cells (IAV or PR8) and metformin-treated cells (IAV + MET or PR8+MET) was measured. The plaque formation rate was calculated using the plaque forming unit of PBS-treated cells as a control. (**F–H)** Total RNA was extracted at each time point. Three types of viral RNA (viral RNA; vRNA, complementary RNA; cRNA, and messenger RNA; mRNA) were measured using strand-specific real-time PCR. For each viral RNA, intracellular viral RNA expression levels were graphed relative to intracellular viral RNA at 2 h post-infection. Data are representative of three independent experiments. The values are expressed as mean ± standard error. Student's t tests were used to compare the groups. *p < 0.05.

The antiviral effect of metformin on IAV in A549 cells infected with IAV was investigated. Cytotoxicity and anti-viral effect on IAV were measured for each metformin concentration (0.2 mM ∼ 5 mM). No cytotoxicity was observed in the 0.2 mM concentration of metformin, and it was confirmed that metformin reduced intracellular viral replication (Fig. 1a and b). In addition, TCID_50_ and plaque assay were performed using the cell culture supernatant, and it was confirmed that virus propagation was reduced with 0.2 mM metformin (Fig. 1c–e). Strand-specific qRT-PCR was conducted on IAV-infected A549 cells to study the intracellular dynamics of IAV replication by metformin. From 24 hpi onwards, v/cRNA synthesis was significantly reduced by metformin but not mRNA synthesis (Fig. 1f–h). As shown in Fig. 1, we confirmed the antiviral effect of metformin on IAV *in vitro* system.

### 2.3 IAV-induced cytokine expression is suppressed by metformin through MAPK/ERK and PI3K/AKT signaling pathways

**Fig. 2:**
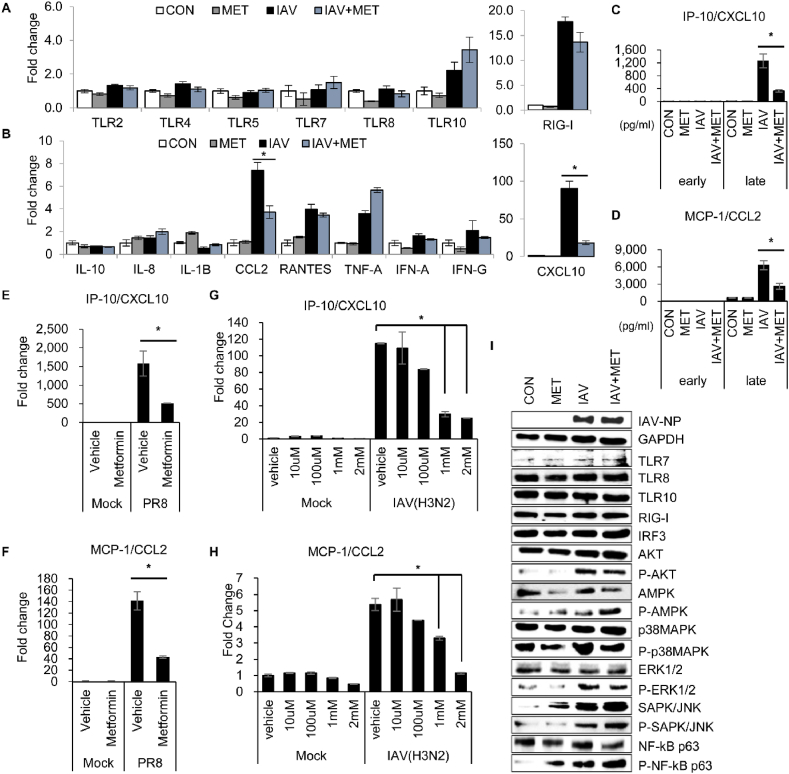
**Influenza A virus-induced IP-10/CXCL10 and MCP-1/CCL2 expression is inhibited by metformin.** THP-1 cells were differentiated into macrophages using 150 nM PMA. Differentiated THP-1 cells were infected with mock or influenza A virus (MOI = 0.1) for 48 h. (**A** and **B)** Total RNA was extracted from the cells and mRNA expression was measured using qRT-PCR. The mRNA expression levels were normalized to GAPDH and expressed as the fold-change in comparison to control. (**C**) IP-10/CXCL10 and (**D**) MCP-1/CCL2 released at 4 h (early) and 48 h (late) after viral infection were measured by immunoassay. The mRNA expression of (**E**) IP-10/CXCL10 and (**F**) MCP-1/CCL2 was confirmed in H1N1(PR8)-infected cells after metformin treatment using qRT-PCR. The mRNA expression levels were normalized to GAPDH. (**G** and **H)** Metformin dose-dependent (**G**) IP-10/CXCL10 or (**H**) MCP-1/CCL2 mRNA levels were measured using qRT-PCR. (**I)** Protein expression related to signaling pathways was confirmed by Western blot. The phosphorylated form was indicated by P, such as P-AKT, P-p38MAPK. GAPDH is shown as the loading control. Data are shown as mean ± standard error of three independent experiments. *p < 0.05. Refer to supplementary file for uncropped version of blots.

**Fig. 3:**
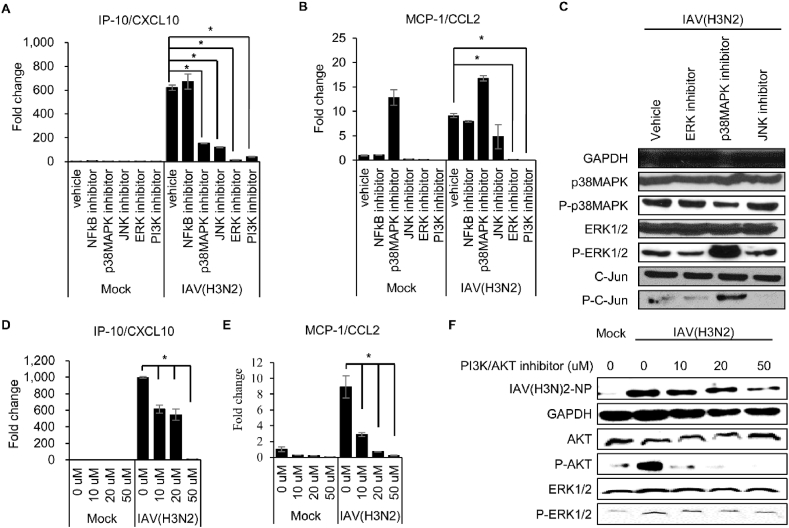
**Influenza A virus-induced IP-10/CXCL10 and MCP-1/CCL2 expression is regulated by AKT/MAPK signaling pathway.** PMA treated THP-1 cells were differentiated. Cells were infected with mock or influenza A virus (MOI = 0.1) and incubated 1 h. Inhibitors including PI3K/AKT inhibitor (LY294002, 30 μM), p38 MAPK inhibitor (SB203580, 10 μM), ERK inhibitor (PD98059, 30 μM), JNK inhibitor (SP600125, 10 μM), and NF-κB inhibitor (SN50, 10 μM) were added while changing the media. After 48 h, total RNA and protein were extracted from the cells. (**A**) IP-10/CXCL10 and (**B**) MCP-1/CCL2 mRNA levels were measured by qRT-PCR and normalized to GAPDH. (**C)** MAPK signaling pathway-related protein expression was confirmed by Western blot. RNA expression of (**D**) IP-10/CXCL10 and (**E**) MCP-1/CCL2 after treatment with PI3K/AKT inhibitor was confirmed using qRT-PCR. (**F)** The effect of the PI3K/AKT specific inhibitor was confirmed by Western blot. Data are shown as mean ± standard error of three independent experiments. *p < 0.05. Refer to supplementary file for uncropped version of blots.

We performed RNA-seq analysis to investigate the mechanism of metformin associated with inhibition of IAV infection. RNA expression patterns between the sample groups were significantly differentiated according to the treatment of metformin rather than IAV infection. The 630 differentially expressed genes (DEGs) were identified in IAV-infected A549 cells treated with metformin and PBS (Supplementary Fig. 1). Functional annotation and gene-set enrichment analysis based on gene ontology (GO) and KEGG database were performed on the DEGs. Several DEGs were involved in host defense mechanisms or the immune system (Supplementary Fig. 1). Luminex assay was performed to measure cytokine secretion, and there was no change due to metformin or the concentration was low, so it was not possible to measure (Supplementary Fig. 2). However, as a result of qRT-PCR, it was confirmed that RNA expression of cytokines such as TNF-α and Interferon gamma inducible protein-10 (IP-10) was reduced by metformin (Supplementary Fig. 2). Western blot results showed that the expression of TLRs and RIG-I, which were increased by viral infection, were significantly reduced by metformin, and the expression of AKT, p38MAPK, ERK and NFκB was also decreased by metformin (Supplementary Fig. 1). Since viral protein expression was also reduced, it is not clear whether this is a direct regulation by metformin or an indirect regulation by viral protein reduction. Because replication of IAV is known to be restricted in monocytes and macrophages, we also investigated the effect of metformin on THP-1 cells infected with IAV. In THP-1 cell study, TLRs expression were not differed by metformin, but IAV-induced monocyte chemoattractant protein-1 (MCP-1/CCL2) and IP-10 expression were significantly reduced by metformin (Fig. 2a–f). The MCP-1 and IP-10 mRNA expression was dose-dependently inhibited by metformin (Fig. 2g and h). In contrast to the results from A549 cells, metformin did not suppress the expression of viral proteins or PRRs in IAV-infected THP-1 cells (Fig. 2i). However, AKT, p38MAPK, and ERK phosphorylation was reduced by metformin in IAV-infected THP-1 cells (Fig. 2i). These results indicate that IAV-induced MCP-1 and IP-10 are regulated by metformin and that the MAPK and AKT signaling pathways may be involved in metformin-activated regulatory mechanisms. Therefore, the mechanisms of regulation of MCP-1 and IP-10 induced by IAV were investigated using the inhibitors. IP-10 and MCP-1 mRNA expression was not inhibited in IAV-infected cells treated with the NF-κB inhibitor, SN50. However, IAV-induced MCP-1 and IP-10 were significantly reduced by PI3K/AKT and ERK inhibitors (Fig. 3a–c). Several studies reported crosstalk between the PI3K/AKT and MAPK/ERK signaling pathways [18,19]. Therefore, we investigated whether ERK inhibition regulates cytokine expression using PI3K/AKT inhibitors. As shown in Fig. 3d–f, ERK phosphorylation and MCP-1 and IP-10 mRNA expression decreased in a dose-dependent manner when treated with PI3K/AKT inhibitors. Therefore, we suggest that the expression of IAV-induced cytokines (MCP-1 and IP-10) and their inhibition by metformin are regulated through the MAPK/ERK and PI3K/AKT pathways.

### 2.4 Influenza-induced atherosclerosis development inhibited by metformin

**Fig. 4:**
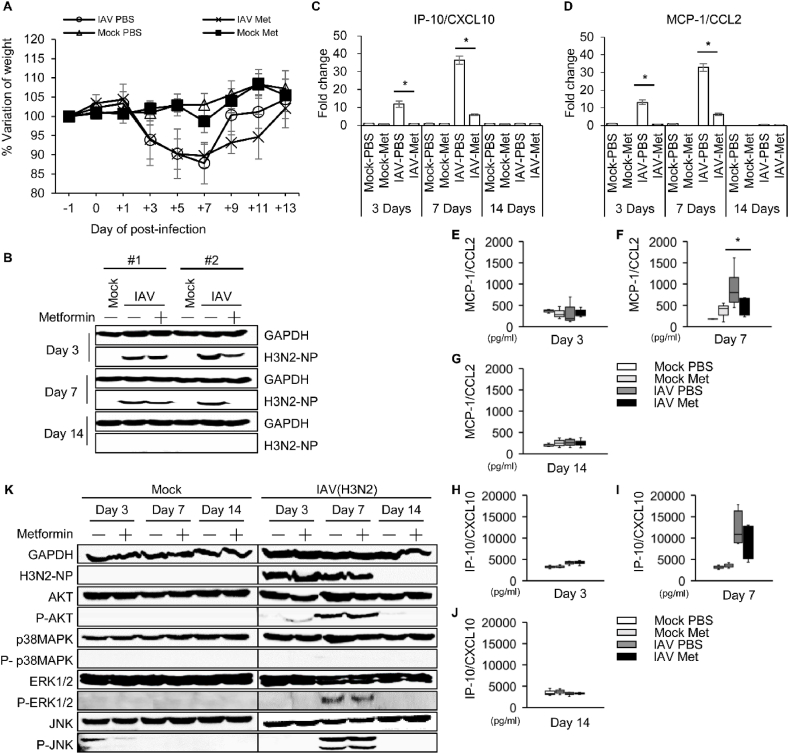
**Virus replication and IP-10/CXCL10 and MCP-1/CCL2 expression in IAV-infected mice are reduced by metformin.** High fat diet-induced atherosclerosis mice were intranasally infected with influenza A virus (Hong Kong/8/1968) and treated with 300 mg/kg/d metformin or vehicle (PBS). **(A)** Body weight was continuously monitored during the study. Mice were euthanized at 3, 7, and 14 d after infection and tissues and blood were collected. (**B)** After protein extraction from lung tissue, GAPDH and viral NP protein were detected by Western blot. GAPDH is shown as a loading control. **(C** and **D)** Total RNA was extracted from mouse lung tissue and (**C**) IP-10/CXCL10 and (**D**) MCP-1/CCL2 expression were measured using qRT-PCR. Target expression levels were normalized to GAPDH. Data are shown as mean ± standard error of three independent experiments. *p < 0.05. (**E-J)** Serum was isolated from blood collected at 3, 7 and 14 days post infection. (**E-G**) MCP-1/CCL2 and (**H-J**) IP-10/CXCL10 in serum isolated from mice was measured using ELISA. Two independent experiments were conducted using serum isolated from the mock infection group (n = 3) and IAV-infection group (n = 4). Data were pooled from two independent experiments. *p < 0.05. (**K)** After protein extraction from lung tissue, GAPDH, viral NP protein and AKT/MAPK signaling related proteins were detected by Western blot. Refer to supplementary file for uncropped version of blots.

**Fig. 5:**
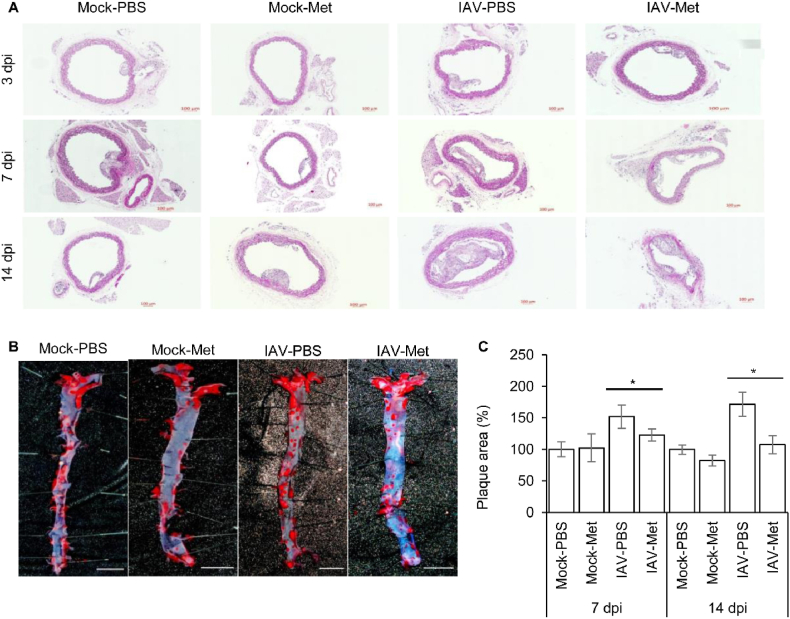
**Atherosclerosis development are reduced by metformin in IAV-infected mice. (A)** Mice aorta sections were stained with hematoxylin and eosin (H&E). Scale bars represent 100 μm. (**B** and **C)** Aorta en-face staining was performed using the aorta isolated from each group of mice at 7 and 14 days post-infection. Atherosclerotic plaques were stained with oil red O and (**B**) showed aorta staining results at 14 days post-infection. Scale bars represent 500 μm. (**C**) Oil red O positive area were quantified using Image J software. Quantitative data are presented as mean ± standard error of three independent experiments. *p < 0.05.

MCP-1 and IP-10 have been implicated in the pathogenesis of cardiovascular diseases. Animal studies using apolipoprotein E-deficient mice (ApoE(−/−)), an atherosclerosis mouse model, were used to determine whether the modulation of IAV-induced MCP-1 and IP-10 by metformin alleviates the underlying disease associated with influenza virus infection. The body weight of the infected mice decreased until 7 days post-infection (dpi) and then recovered (Fig. 4a). As shown in Fig. 4b and k, viral proteins peaked at 7 dpi in the lung tissue of IAV-infected mice. The expression of viral protein was lower in the metformin-treated infection group than in the PBS-treated infection group (Fig. 4b). Additionally, in histological analysis, virus peaked at 7 dpi in the lung of infection group (IAV-PBS and IAV-Met) consistent with the Western blot results (Supplementary Figure 3). As a results of H&E staining, the influx of immune cells into the lungs of the metformin-treated infection group (IAV-Met) was reduced compared to the PBS-treated infection group (IAV-PBS) at 3 and 7 dpi (Supplementary Figure 3). MCP-1 and IP-10 mRNA expression was significantly increased in the lungs of IAV-infected mice, and that MCP-1 and IP-10 mRNA were dramatically decreased by metformin (Fig. 4c and d). MCP-1 and IP-10 levels in mouse serum were quantitatively analyzed using enzyme-linked immunosorbent assay (ELISA). No significant difference was observed between the groups at 3 or 14 dpi (Fig. 4e–j). On day 7 post-infection, MCP-1 and IP-10 secretion in the IAV-PBS group increased dramatically. This increase was suppressed in the IAV-Met group (Fig. 4f and i). Particularly, MCP-1 secreted into serum was significantly decreased in the IAV-Met group (426.90 ± 224.38 pg/ml) compared to the IAV-PBS group (895.57 ± 450.94 pg/ml). The protein expression related to the AKT and MAPK signaling pathways involved in the secretion of MCP-1 and IP-10 confirmed in the *in vitro* studies was analyzed in mouse lung tissue by Western blot (Fig. 4k). On day 7 post-infection, the total protein expression of AKT and p38MAPK was decreased by metformin in the lungs of mice in the IAV-infected group, but there was no difference in the mock-infected group. In addition, ERK phosphorylation was slightly decreased by metformin in the IAV-infected group at 7 dpi. These results indicated that MCP-1 and IP-10 expression was regulated through the AKT and MAPK signaling pathway. We next investigated whether reduced MCP-1 and IP-10 secretion influenced the pathogenesis of atherosclerosis in the mouse aorta. H&E staining showed that the development of atherosclerosis significantly increased due to IAV infection, compared with the Mock-infected groups on day 14 post-infection (Fig. 5a). The development of atherosclerosis was significantly decreased in the IAV-Met group at 14 dpi compared to the IAV-PBS group (Fig. 5a). Aorta en-face staining was performed to quantitatively analyze atherosclerosis due to IAV infection and metformin treatment. Tissues were stained with Oil Red O and analyzed for atherosclerotic lesions. As shown in Fig. 5b and c, atherosclerotic lesions were increased in the IAV-PBS group (1.71 fold, p-val = 0.0036) compared to the Mock-PBS groups. The IAV-induced atherosclerotic lesions were suppressed in the IAV-Met group similarly to the mock-infected group (Mock-PBS or Mock-Met). Interestingly, the inhibitory effect of metformin on the development of atherosclerosis was not observed in the mock infection groups. These findings indicate that metformin alleviates the acutely exacerbated atherosclerosis cause by IAV, but does not cure pre-existing atherosclerosis. Our findings also suggest that the alleviation effect of metformin is related to MCP-1 and IP-10 and that these cytokines are regulated through the AKT and MAPK signaling pathway.

## 3 Discussion

This study was conducted to investigate the drug repositioning potential of metformin in patients with underlying diseases that are at high risk of death from IAV infection. In statistical analysis, we found that metformin administration reduced IAV-related deaths by 62% in DM patients. These results are consistent with previous studies that metformin reduces cardiovascular risk and provides health benefits for people with DM. Recently, it was reported that metformin was significantly associated with a decrease in mortality in obese or type 2 diabetic women hospitalized for COVID-19 [20]. As described above, several studies have reported that metformin can reduce the risk of infectious diseases and complications in DM patients, but studies on non-DM patients are insufficient [[20], [21], [22]]. In this study, the antiviral and immunomodulatory effects of metformin were investigated in non-DM conditions through *in vitro* and *in vivo* studies. As shown in Fig. 1, Fig. 4, virus replication and proliferation were slightly reduced by metformin. In the serum of IAV-infected ApoE(−/−) mice and culture medium of IAV-infected THP-1 cells, the cytokine reduction by metformin was confirmed, and the immunomodulatory effect of metformin on IAV infection was demonstrated. In particularly, MCP-1 and IP-10 expression was dramatically reduced by metformin treatment in IAV-infected cells. MCP-1 and IP-10 are considered as key factors in the immunomodulatory effect of metformin on IAV infection. MCP-1 and IP-10 recruit monocytes/macrophages, T cells and dendritic cells to the sites of inflammation. MCP-1 and IP-10 are known to be associated with various pathologies including atherosclerosis, cardiovascular disease, rheumatoid arthritis, and viral infection [23,24]. A recent COVID-19 study report showed that IP-10 and MCP-1 were overexpressed in patients with COVID-19, and cardiovascular disease was reported in up to 20% of patients with COVID-19 [25]. Recently, the importance of the NLRP3 inflammasome in cardiovascular disease-related inflammation has been extensively studied, and it is known that NLRP3 inflammasome increases the risk of cardiovascular disease by increasing the activation and release of IL-1 family cytokines [26]. In addition, various studies have suggested that metformin inhibits NLRP3 inflammasome activity, thereby suppressing IL-1beta secretion and reducing inflammation [27,28]. However, we did not confirm significant changes in the mRNA expression of IL-1beta by metformin treatment in IAV-infected cells, but the expression of MCP-1 and IP-1 was significantly decreased (Fig. 2 and Supplementary Fig. 2). Therefore, we considered that metformin mitigates the exacerbation of cardiovascular disease by regulating excessive immune cell influx in IAV-related cardiovascular events. In the ApoE(−/−) mice study, we demonstrated the alleviation effect of metformin in exacerbation of IAV-induced atherosclerosis. In conclusion, we suggested that the acute exacerbation of cardiovascular disease due to IAV infection is ameliorated by the immunomodulatory effect of metformin, which modulates IAV-induced cytokines such as MCP-1 and IP-10. Metformin is known to regulate cellular functions by various mechanisms of action, so the immunomodulatory mechanism of action against influenza virus was studied through an *in vitro* study. RNA-seq analysis revealed 630 genes whose expression was regulated by metformin in IAV-infected cells. As a result of KEGG analysis, there were 19, 18, and 16 genes related to the MAPK signaling pathway, the PI3K-AKT signaling pathway, and the cytokine-cytokine receptor interaction (Supplementary Table 1). In particular, the receptor genes (PDGFRA, ADORA1, TLR5) involve in upstream of the signaling pathway were down-regulated, and genes involved in the downstream were regulated to inhibit the signaling pathway. The up-regulated genes (DUSP1, DUSP8, JUN, NFKB2) and the down-regulated genes (MAP2K6, SYK, HSPA1A, HSPA1B) were identified among the downstream related genes of the cell signaling pathways (Supplementary Table 1). DUSP1 and DUSP8 are known to inactivate MAPK signaling pathway through dephosphorylation of the MAPK superfamily [29]. Interestingly, there is a report that activation of ERK1/2 was increased by acute pathological stress stimulation of the heart in DUSP8 gene knockout mice [30]. MAP2K6 is a member of the dual specificity protein kinase family, which functions as an ERK kinase in response to inflammatory cytokine or environmental stress. In addition, there is a report that MAP2K6 is an important mediator for TNF-α-mediated MCP-1 expression in endothelial cells.34 SYK mediate a variety of cellular responses through direct binding with signaling intermediates, including PLCγ, and PI3K [31]. PI3K/AKT signaling pathway and MAPK signaling pathway are activated by IAV infection and are known to regulate a variety of cellular responses including cell survival, cell proliferation and cytokine expression. In addition, several studies have reported that there is cross talk between P3IK/AKT and MAPK signaling [18,19]. In this study, we confirmed a dose-dependent decrease in ERK activity with the concentration of an AKT inhibitor, indicating a crosstalk between PI3K/AKT and MAPK/ERK. We demonstrated that the activation of PI3K/ATK signaling pathway and MAPK signaling pathway induced by IAV were regulated by metformin. The expression of PRRs such as TLR and RIG-I, which are proteins upstream of the signaling pathway, was significantly increased in virus-infected A549 cells, but there was no significant change in THP-1 cells. Increased expression of PRRs in IAV-infected A549 cells was significantly inhibited by metformin. IAV replication and propagation are known to be decreased in monocyte-derived macrophage compared to A549 cells. We observed an increase in viral protein expression over time in infected A549 cells, but no change in viral protein expression over time in THP-1 cells (Supplementary Fig. 4). Therefore, it is considered that the difference in expression of PRRs compared in IAV-infected A549 cells and IAV-infected THP-1 cells is due to virus susceptibility to each cell. Unlike PRRs, expression and activation of downstream proteins including ERK, p38, JNK, and AKT were similarly observed in both cells and mouse lung tissue (Fig. 4k). Consequently, we suggested that immunomodulatory effect of metformin on IAV infection is regulated through the AKT/MAPK signaling pathway. We consider that downstream proteins play a more important role than receptor proteins such as TLR and RIG-I in the regulation of MCP-1 and IP-10 by metformin.

In conclusion, we demonstrated the potential and mechanism of action of metformin as a treatment for cardiovascular disease exacerbation caused by IAV infection. Although this study is limited to the study of the effect of metformin administration after viral infection, it is a meaningful study confirming the repositioning potential of metformin as a therapeutic agent for exacerbation of cardiovascular disease caused by IAV infection. For clinical application, further studies such as the effect of metformin according to the time of treatment including administration before viral infection are needed.

## 4 Materials and methods

### 4.1 Statistical analysis

Clinical information of enrolled patient with influenza-like illness (ILI) during the 2011–2019 was collected through the Hospital-based Influenza Morbidity and Mortality (HIMM) surveillance system. Among the patients enrolled in HIMM, data were collected from DM patients (n = 2378). The collected data include medical and pharmacy information, laboratory results, and enrolment records. Patients with missing data were excluded, and statistical analysis was performed by collecting data for a total of 645 patients. Influenza-related death was categorized as deaths within 30 days of influenza virus detection. In DM patients, we analyzed the association between influenza-related death and various factors such as the patient's age, underlying disease, vaccination and medications. We did logistic regression analysis of retrospective study data. The independent variable was influenza related death. The age, level of blood glucose, and number of underlying diseases were expressed as median ± standard deviation. Other categorical variables are represented as binary variables. First, the correlation analysis was performed between the influenza related death and other variables. Considering the effect between the dependent variables, the relation between the total dependent variable and influenza-related death was analyzed using logistic regression analysis. This study's protocol was approved by the Institutional Review Board of Korea University Guro Hospital (2011GR0214 and 2017GR0172). Statistical analysis were performed using SPSS program. Statistical significance was defined as *p* < 0.05.

### 4.2 Animal study

Six-week old ApoE(−/−) mice were fed an atherogenic diet for 12 weeks. Atherosclerotic mice (n = 48) were anesthetized with a mixture of alfaxalone and xylazine. The study group was divided into four groups: Mock-PBS, Mock-MET, IAV-PBS, and IAV-MET. Influenza A virus infection groups (n = 30; IAV-PBS and IAV-MET) were inoculated intranasally with 10^3^ TCID_50_ of influenza A virus (HK/68). Non-infection groups (n = 18; Mock-PBS and Mock-MET) were intranasally inoculated with PBS in equal volumes as the infected group. Metformin treatment groups were fed 300 mg/kg/d metformin through the drinking water. Body weight was measured until the end of the experiment. Mice were euthanized at 3, 7, and 14 d post-infection. The mice were sacrificed using air saturated with CO_2_. The extracted tissue was placed in saline buffer and stored at 4 °C. Serum was separated from blood using centrifugation and stored at −80 °C. The protocol was approved by the Animal Welfare Committees of the Laboratory Animal Research Center of the Korea University College of Medicine (KOREA-2020-0197).

### 4.3 Cell and virus

A549 cells were cultured in Minimum Essential Medium (MEM) in 5% CO2 at 37 °C. THP-1 cells were cultured in Roswell Park Memorial Institute medium 1640 (RPMI-1640). THP-1 cells were differentiated into macrophages by 72 h incubation with 150 nM phorbol 12-myristate 13-acetate (PMA). The influenza A virus used in the experiment was A/Hong Kong/8/1968(H3N2) strain (HK/68) and A//(H1N1) strain (PR8).

### 4.4 Quantitative real-time polymerase chain reaction (qRT-PCR)

Total RNA was isolated using Trizol reagent according to the manufacturer's instructions. cDNA was synthesized using the PrimeScript 1st strand complementary cDNA synthesis kit (Takara Bio Inc, Shiga, Japan) at 42 °C for 1 h. All qRT-PCR were carried out using an ABI QuantStudio 6 Flex (Applied Biosystems Inc., Foster City, CA) under the following conditions: 10 min at 95 °C followed by 40 cycles of 95 °C for 15 s, 60 °C for 1min. Quantification of target gene was normalized with GAPDH as an internal control.

### 4.5 Western blot and immunoassay

RIPA buffer was used to lyse cells and tissues according to the manufacturer's instructions. The lysate was loaded on SDS-PAGE and then the proteins are separated by electrophoresis. The separated protein was electro-transferred to the nitrocellulose membrane. The protein expression level shown using primary antibodies and secondary antibodies: anti-influenza NP, anti-TLR7, anti-TLR8, anti-TLR10, anti-RIG-I, anti-IRF3, anti-AKT, anti-phospho-AKT, anti-AMPK, anti-phospho-AMPK, anti-p38 MAPK, anti-phospho-p38 MAPK, anti-ERK, anti-phospho-ERK, anti-JNK, anti-phospho-JNK, anti-NFkBp63, anti-phospho-NKkBp63, anti-GAPDH, and horseradish peroxidase-conjugated goat anti-rabbit antibody or goat anti-mouse antibody. Human cytokines were measured in supernatant of cell cultures using the Human XL Cytokine Discovery Base Kit (R&D Systems, Minneapolis, USA) according to the manufacturer's instructions. In mouse serum, levels of MCP-1 and IP-10 were quantified using a mouse MCP-1(R&D Systems, Minneapolis, USA) or mouse IP-10 (NOVUS Biologicals, Abingdon, UK) ELISA kit, according to the manufacturer's instructions. After reaction, immediately read at 450 nm. The mean absorbance for each set of duplicate samples and standards was calculated.

### 4.6 Tissue staining and en-face analysis

Isolated lung and aorta were fixed with 10% neutral-buffered formalin for 24 h at room temperature. The fixed tissues were embedded in paraffin. The paraffin-embedded tissue was cut into sections and mounted on poly-*l*-lysine coated slides. A prepared slide of each tissue was stained with hematoxylin and eosin (H&E) or rabbit anti-hemagglutinin (HA) polyclonal antibody (GeneTex, Irvine, CA). The slides were incubated with detection was performed by a rabbit-specific HRP/DAB detection kit (Abcam, Cambridge, UK), according to the manufacturer's instructions. Mayer's hematoxylin (Sigma-Aldrich, St. Louis, MO) was used as a counterstain. Slides were observed under a ZEISS Axioscan. En-face analysis of aorta tissue was performed as previously described [32]. The images of stained aorta were analyzed using Image J software.

## Author contribution statement

Han Sol Lee: Conceived and designed the experiments; Performed the experiments; Contributed reagents, materials, analysis tools or data; Wrote the paper. Woo Joo Kim: Conceived and designed the experiments; Contributed reagents, materials, analysis tools or data; Wrote the paper. Ji Yun Noh, Joon Young Song, Hee Jin Cheong: Analyzed and interpreted the data.

## Data availability statement

Data will be made available on request.

## Declaration of competing interest

The authors declare that they have no known competing financial interests or personal relationships that could have appeared to influence the work reported in this paper.
