# Supplementary material for: Metformin reduces the risk of developing influenza A virus related cardiovascular disease
Source: Heliyon. 2023 Sep 21;9(10):e20284. doi: 10.1016/j.heliyon.2023.e20284 (PMC10556598; doi:10.1016/j.heliyon.2023.e20284)

Figure 2.I

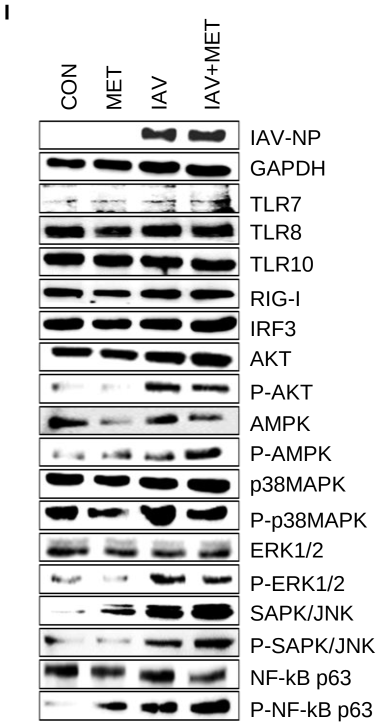

Figure 2.I uncropped membrane

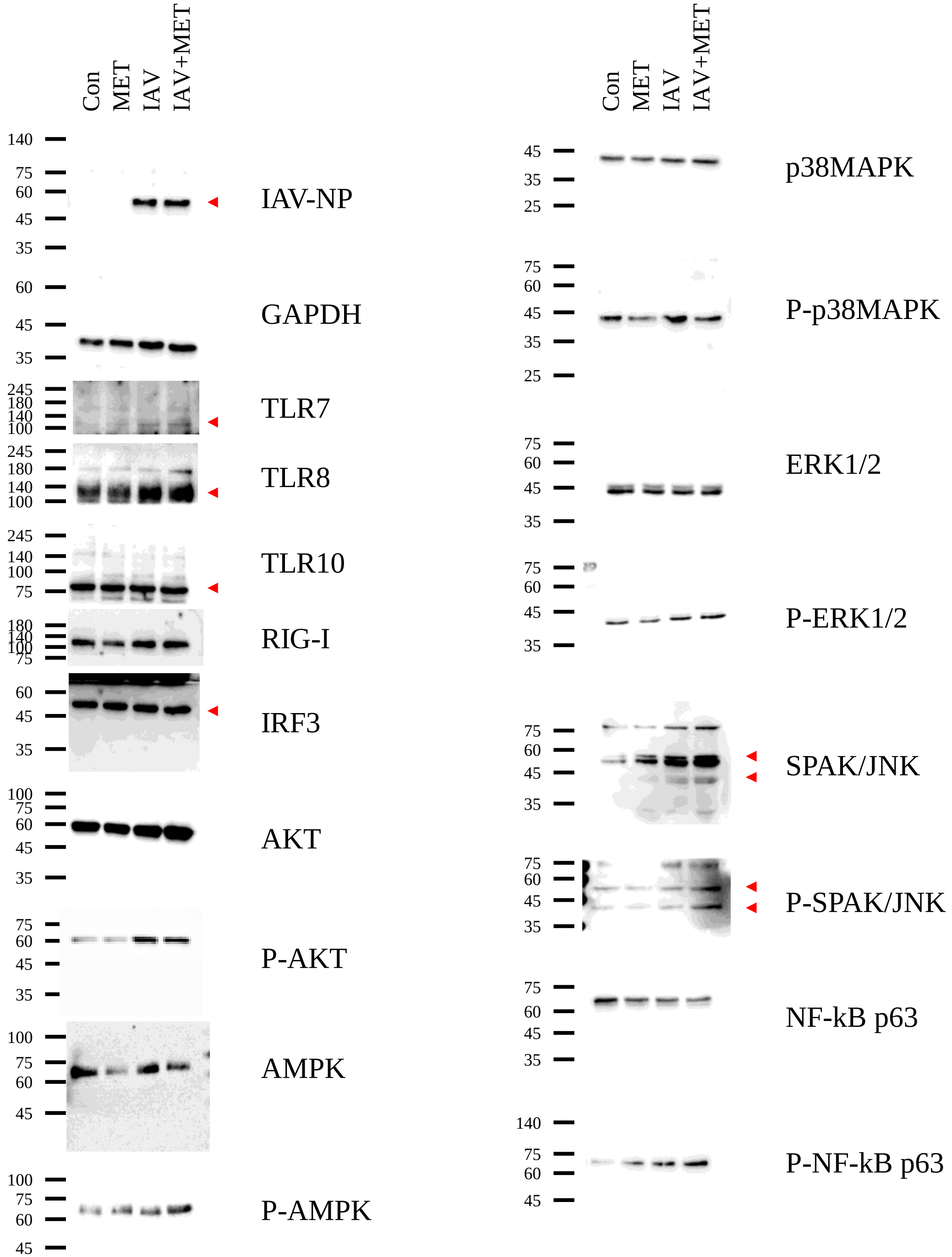

Figure 3.C uncropped membrane

Figure 3.C

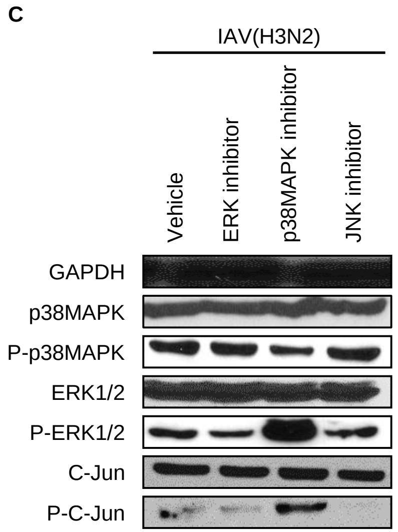

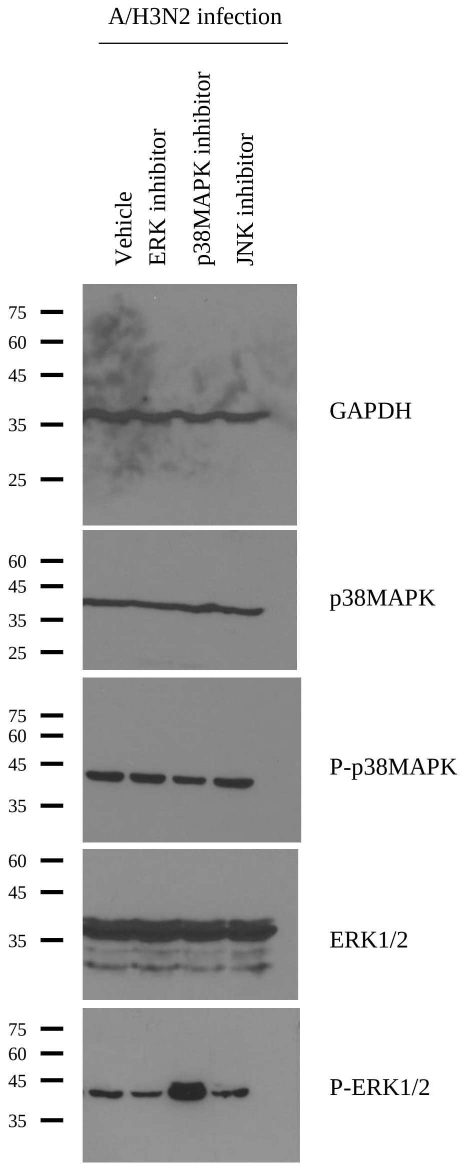

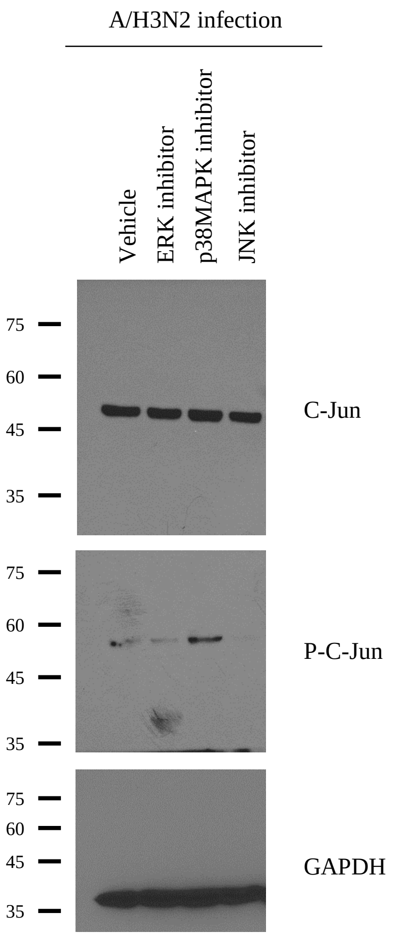

Figure 3.F

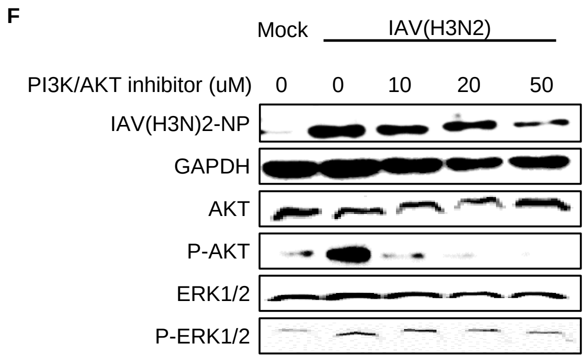

Figure 3.F uncropped membrane

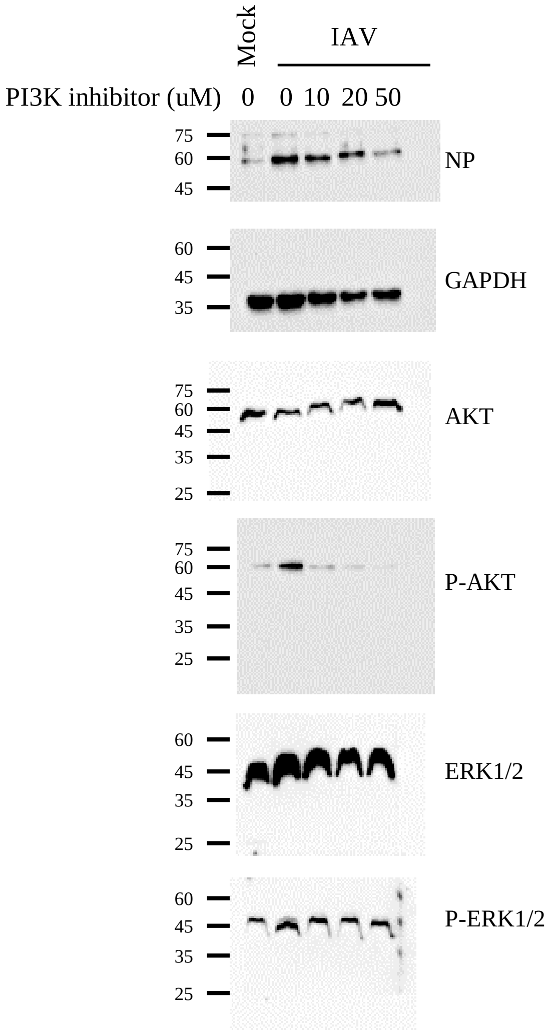

Figure 4.B uncropped membrane

Figure 4.B

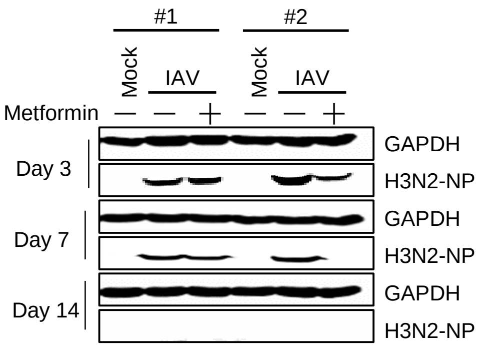

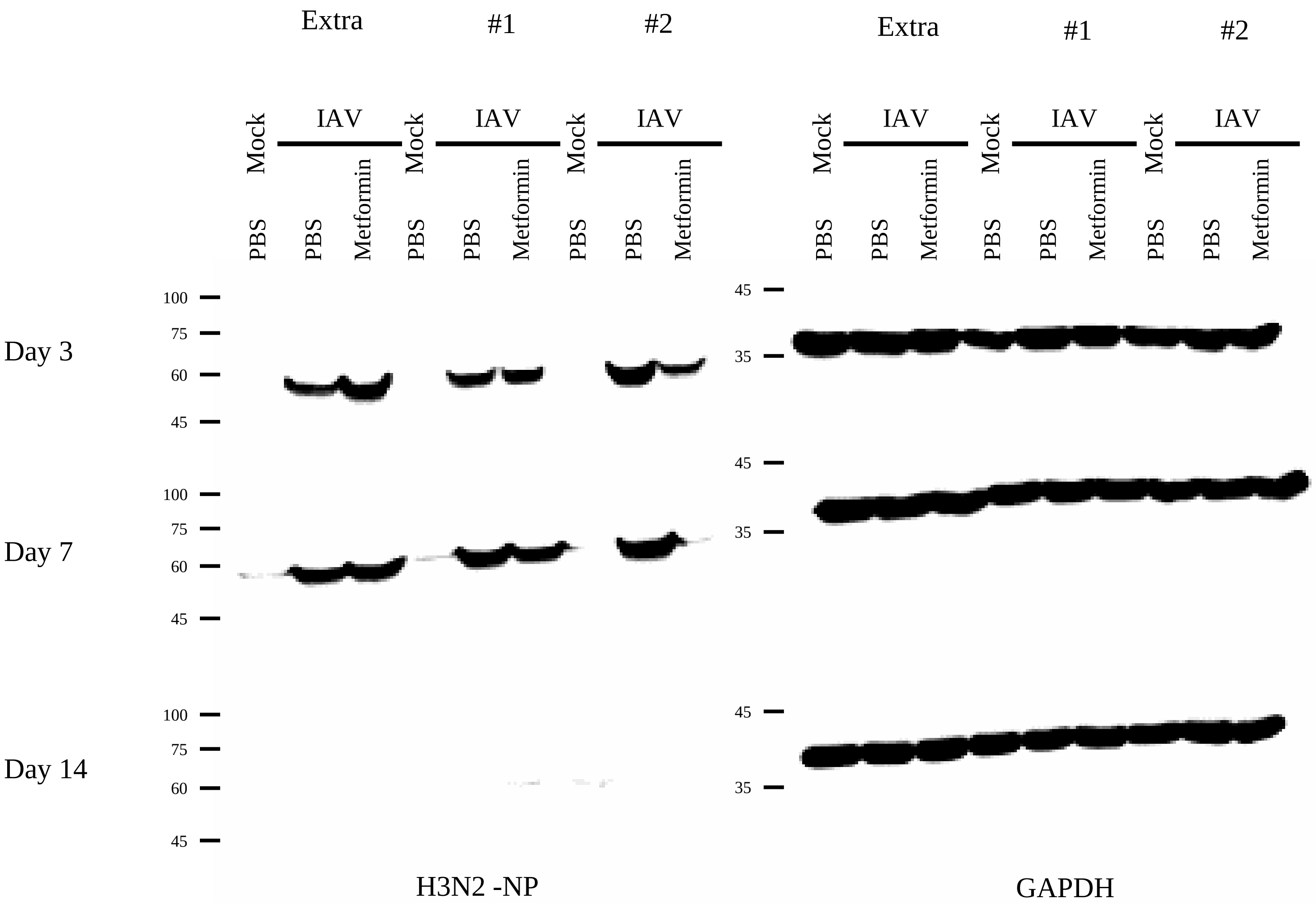

Figure 4.K

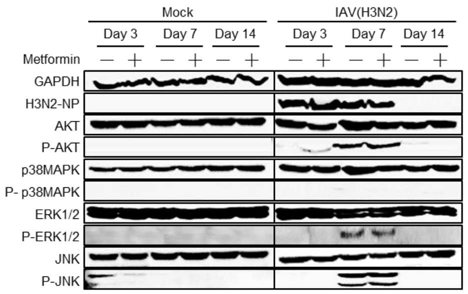

Figure 4.K uncropped membrane

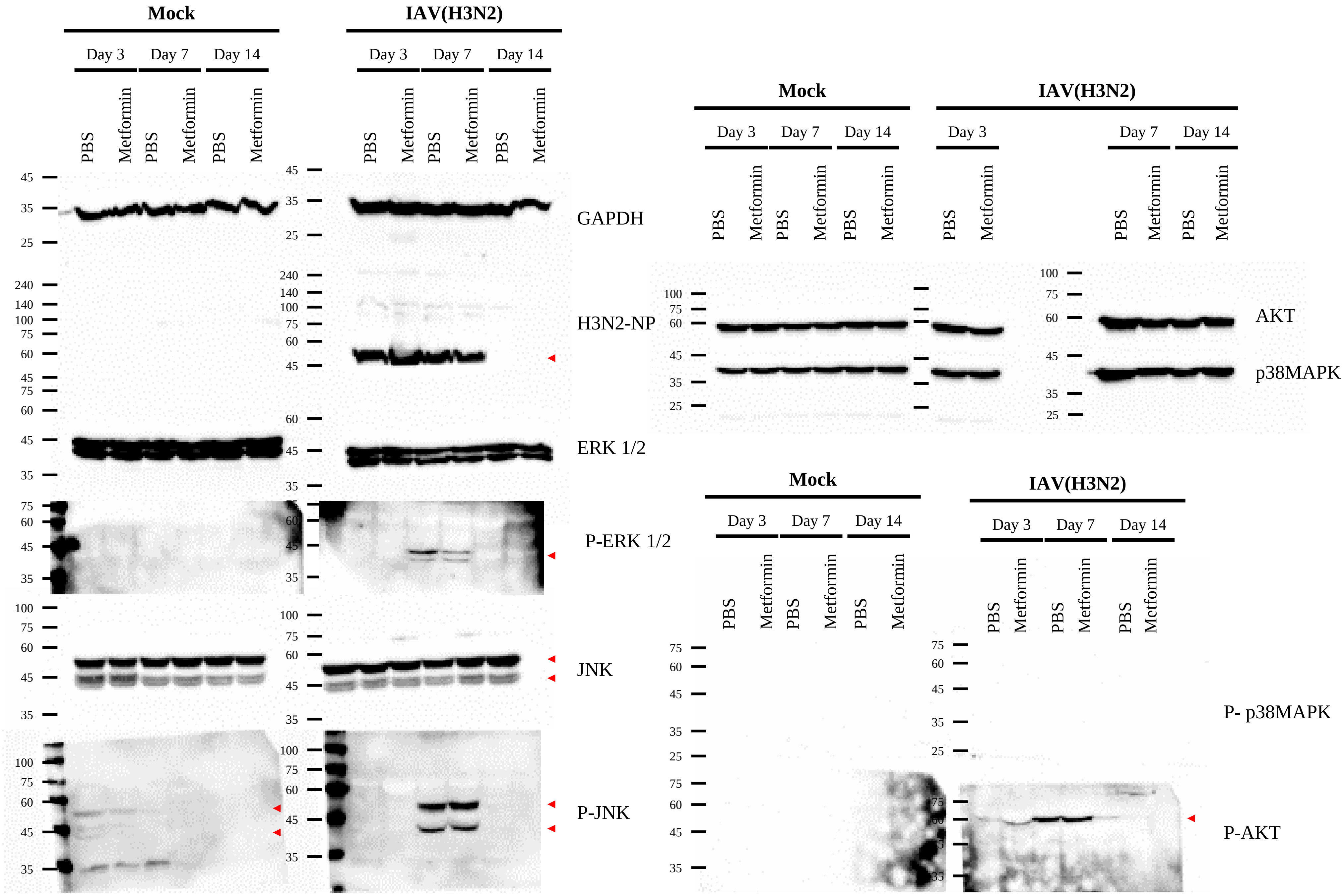

Supplemental Figure 1.G

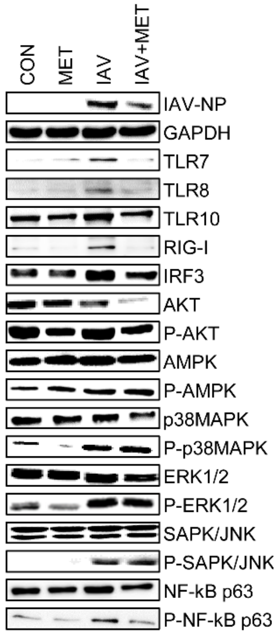

Supplemental Figure 1.G uncropped membrane

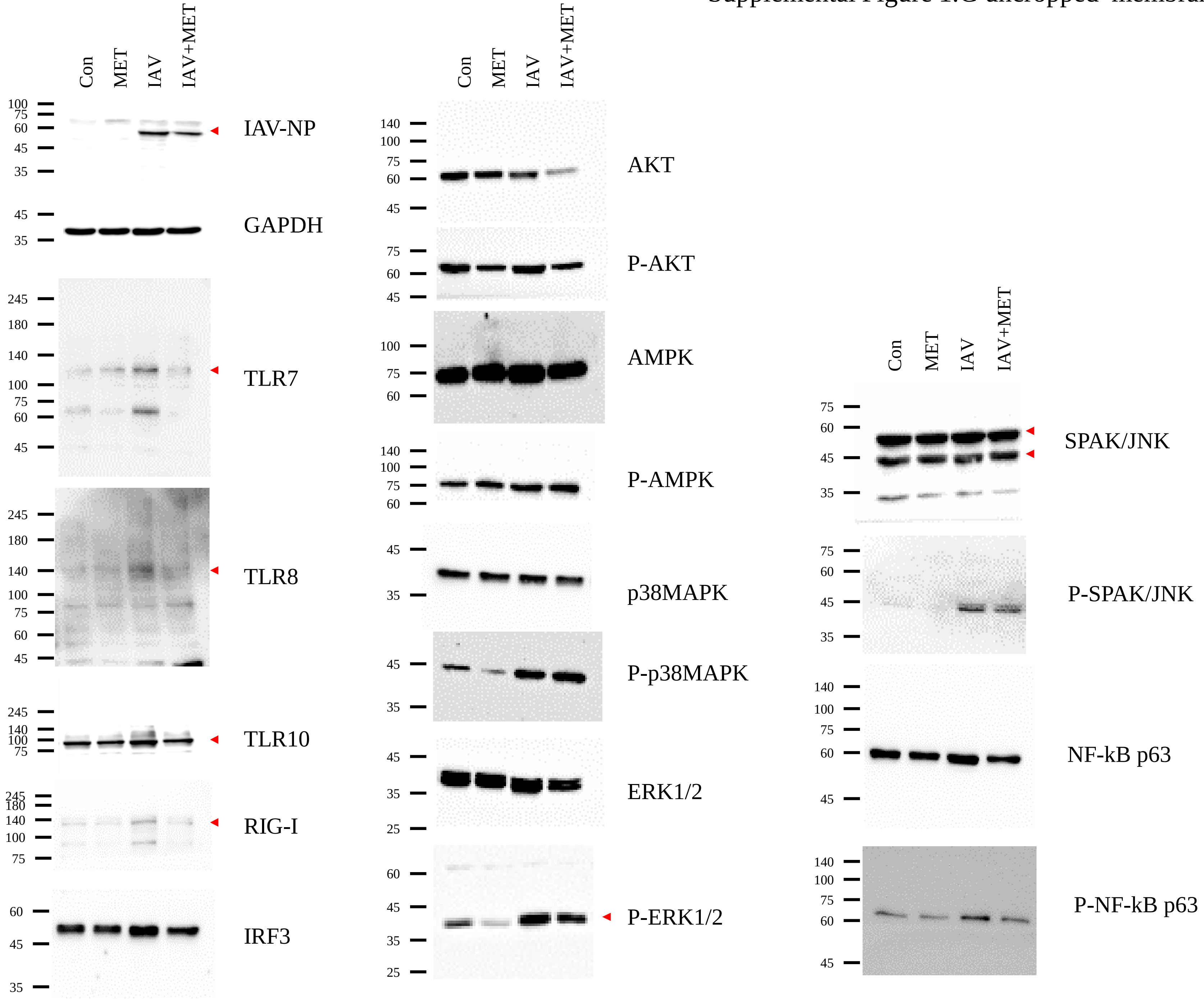

Supplemental Figure 4

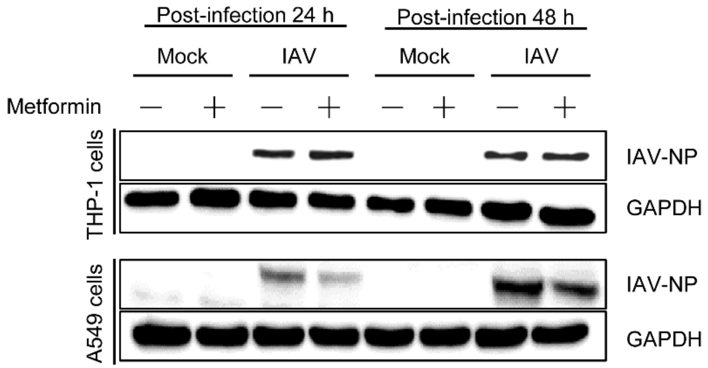

Supplemental Figure 4 uncropped membrane

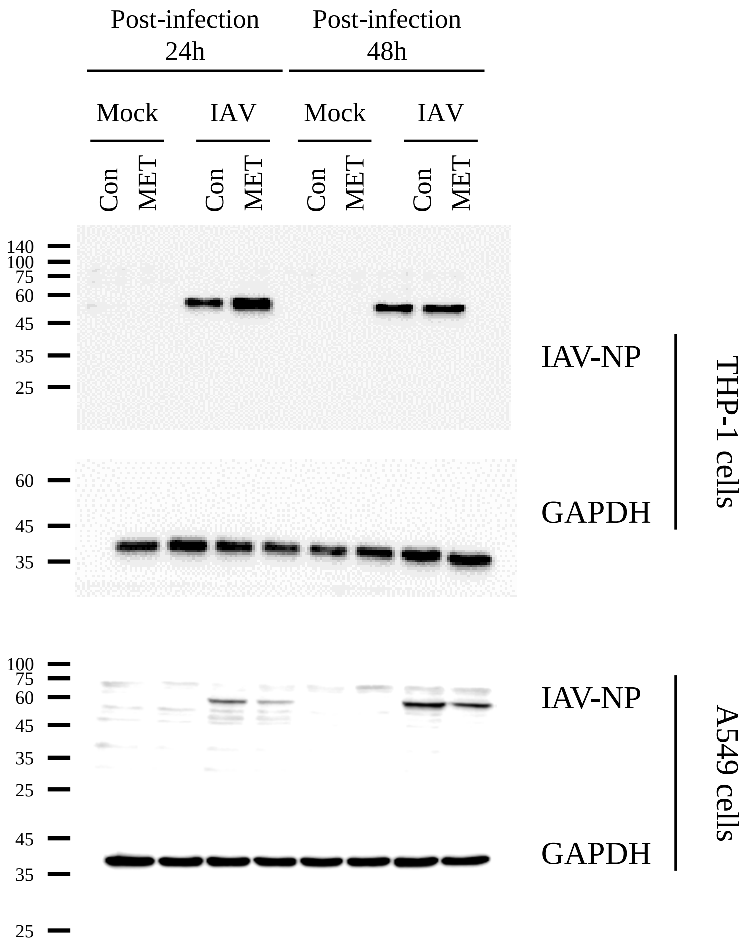

Supplement: Multimedia component 1 [file mmc1.pdf]
