# Supplementary material for: Metformin reduces the risk of developing influenza A virus related cardiovascular disease
Source: Heliyon. 2023 Sep 21;9(10):e20284. doi: 10.1016/j.heliyon.2023.e20284 (PMC10556598; doi:10.1016/j.heliyon.2023.e20284)
Supplement: Multimedia component 2 [file mmc2.docx]

**Supplemental Table 1. The results of Pathway map enrichment analysis for the comparison combination of IAV and IAV-MET**

| MapID | MapName | Number of Significant Genes | P-value | FDR | Genes |
| --- | --- | --- | --- | --- | --- |
| 05165 | Human papillomavirus infection | 20 | **4.85298E-09** | **7.17169E-07** | LAMB4, ATP6V0D2, HES1, TNC, IRF1, ITGB7, ITGB8, LAMA4, MX1, NOTCH3, ATP6V0A4, WNT4, HES4, VEGFA, WNT1, HES7, ITGA10, OASL, CREB5, ISG15 |
| 04010 | MAPK signaling pathway | 19 | **5.33211E-09** | **7.17169E-07** | GADD45A, DDIT3, DUSP1, DUSP8, EREG, HSPA1A, HSPA1B, IL1RAP, JUN, AREG, GADD45B, NFKB2, NGF, PDGFRA, MAP2K6, TGFB2, VEGFA, CACNA1G, FGF19 |
| 01100 | Metabolic pathways | 42 | **1.90937E-08** | **1.2932E-06** | NT5C1B-RDH14, ST20-MTHFS, B3GALT5, CHKB, GALNT5, GALNT15, CMPK2, ADH6, NUDT16, CYP1A1, CYP3A5, DDC, LDHD, ALDOC, ACSBG1, ATP6V0D2, GAD1, ANPEP, C1GALT1C1, DMGDH, HK2, AOC2, HSD11B2, HYAL1, MAT1A, RRM2B, ATP6V0A4, PDE1A, SPTLC3, OGDHL, BAAT, PTGS1, CARNS1, UPP1, CA9, HKDC1, PLCD4, PDE8B, AOC3, GALNT4, DHRS3, GDA |
| 05200 | Pathways in cancer | 24 | **1.92298E-08** | **1.2932E-06** | LPAR6, EGLN3, GADD45A, AGT, EDNRA, F2, LAMB4, BBC3, HES1, HSP90AA1, IL6, CXCL8, JUN, LAMA4, GADD45B, NFKB2, NOTCH3, PDGFRA, WNT4, TGFB2, VEGFA, WNT1, CXCR4, FGF19 |
| 04668 | TNF signaling pathway | 12 | **6.59492E-08** | **3.54807E-06** | CEBPB, IL6, IRF1, JUN, JUNB, LTA, MAP2K6, BCL3, CCL5, CCL20, SOCS3, CREB5 |
| 04151 | PI3K-Akt signaling pathway | 18 | **3.7735E-07** | **1.69178E-05** | SGK2, LPAR6, EREG, LAMB4, HSP90AA1, TNC, IL6, ITGB7, ITGB8, AREG, LAMA4, NGF, PDGFRA, SYK, VEGFA, ITGA10, CREB5, FGF19 |
| 04060 | Cytokine-cytokine receptor interaction | 16 | **9.0775E-07** | **3.48835E-05** | CCR6, ACVR1C, IL34, IL1RAP, IL6, CXCL8, IL11, LTA, NGF, CCL5, CCL20, TGFB2, IL1R2, CXCR4, GDF15, TNFSF15 |
| 05202 | Transcriptional misregulation in cancer | 13 | **1.16059E-06** | **3.54157E-05** | CEBPB, GADD45A, DDIT3, HPGD, IGFBP3, IL6, CXCL8, ITGB7, MAF, GADD45B, CDK14, IL1R2, NR4A3 |
| 05323 | Rheumatoid arthritis | 10 | **1.18491E-06** | **3.54157E-05** | ATP6V0D2, IL6, CXCL8, IL11, JUN, ATP6V0A4, CCL5, CCL20, TGFB2, VEGFA |
| 04080 | Neuroactive ligand-receptor interaction | 16 | **4.99949E-06** | **0.000134486** | LPAR6, CALCRL, ADM, ADORA1, ADRA2B, GPR156, AGT, EDNRA, F2, GRIK5, INSL3, MTNR1A, NPY2R, P2RY6, CHRNA10, RLN2 |
| 04657 | IL-17 signaling pathway | 9 | **1.13008E-05** | **0.000276357** | CEBPB, FOSB, HSP90AA1, IL6, CXCL8, JUN, LCN2, CCL20, FOSL1 |
| 04510 | Focal adhesion | 12 | **1.30709E-05** | **0.000280199** | VAV3, LAMB4, TNC, ITGB7, ITGB8, JUN, LAMA4, PAK3, PDGFRA, SHC3, VEGFA, ITGA10 |
| 04380 | Osteoclast differentiation | 10 | **1.35412E-05** | **0.000280199** | FOSB, JUN, JUNB, NFKB2, MAP2K6, SYK, TGFB2, FOSL1, SOCS1, SOCS3 |
| 04933 | AGE-RAGE signaling pathway in diabetic complications | 9 | **1.71994E-05** | **0.000330474** | AGT, EGR1, IL6, CXCL8, JUN, SERPINE1, TGFB2, VEGFA, PLCD4 |
| 05034 | Alcoholism | 11 | **3.71504E-05** | **0.000601485** | DDC, FOSB, GNAO1, SHC3, PKIA, HIST1H2AC, HIST1H2BN, HIST1H4K, HIST1H4J, HIST1H4H, CREB5 |
| 05224 | Breast cancer | 10 | **3.8012E-05** | **0.000601485** | GADD45A, HES1, JUN, GADD45B, NFKB2, NOTCH3, SHC3, WNT4, WNT1, FGF19 |
| 05145 | Toxoplasmosis | 9 | **3.6907E-05** | **0.000601485** | LAMB4, GNAO1, HSPA1A, HSPA1B, LAMA4, LDLR, MAP2K6, TGFB2, SOCS1 |
| 05203 | Viral carcinogenesis | 11 | **7.52922E-05** | **0.0011252** | ATP6V0D2, GTF2A1, IRF7, JUN, NFKB2, SYK, HIST1H2BN, HIST1H4K, HIST1H4J, HIST1H4H, CREB5 |
| 04061 | Viral protein interaction with cytokine and cytokine receptor | 8 | **0.000127987** | **0.001812027** | CCR6, IL34, IL6, CXCL8, LTA, CCL5, CCL20, CXCR4 |
| 05206 | MicroRNAs in cancer | 13 | **0.000147052** | **0.001883662** | CYP1B1, TNC, MIR103A2,MIR23A, MIR23B, MIR27A, MIR27B, NOTCH3, PDGFRA, TGFB2, VEGFA, TP63, SOCS1 |
| 04915 | Estrogen signaling pathway | 9 | **0.000146971** | **0.001883662** | HBEGF, GNAO1, HSPA1A, HSPA1B, HSP90AA1, JUN, SHC3, KRT20, CREB5 |
| 04115 | p53 signaling pathway | 7 | **0.000161135** | **0.001970238** | GADD45A, BBC3, IGFBP3, GADD45B, RRM2B, SERPINE1, SESN2 |
| 04930 | Type II diabetes mellitus | 6 | **0.000183406** | **0.002145055** | HK2, MAFA, HKDC1, SOCS1, CACNA1G, SOCS3 |
| 04066 | HIF-1 signaling pathway | 8 | **0.00021154** | **0.002371007** | EGLN3, ALDOC, HK2, IL6, SERPINE1, TF, VEGFA, HKDC1 |
| 04390 | Hippo signaling pathway | 9 | **0.000298964** | **0.003216855** | CTGF, WWTR1, BBC3, AREG, SERPINE1, WNT4, SNAI2, TGFB2, WNT1 |
| 05169 | Epstein-Barr virus infection | 10 | **0.000362356** | **0.003748996** | GADD45A, HES1, IL6, IRF7, JUN, GADD45B, NFKB2, MAP2K6, SYK, ISG15 |
| 05210 | Colorectal cancer | 7 | **0.000396604** | **0.003951354** | GADD45A, EREG, BBC3, JUN, AREG, GADD45B, TGFB2 |
| 05134 | Legionellosis | 6 | **0.000460615** | **0.00442519** | HSPA1A, HSPA1B, IL6, CXCL8, NFKB2, TLR5 |
| 05410 | Hypertrophic cardiomyopathy (HCM) | 7 | **0.000498213** | **0.004621353** | AGT, IL6, ITGB7, ITGB8, SLC8A1, TGFB2, ITGA10 |
| 05163 | Human cytomegalovirus infection | 10 | **0.000789308** | **0.006835942** | GNAO1, TMEM173, IL6, CXCL8, PDGFRA, MAP2K6, CCL5, VEGFA, CXCR4, CREB5 |
| 05162 | Measles | 8 | **0.000813198** | **0.006835942** | BBC3, HSPA1A, HSPA1B, IL6, IRF7, JUN, MX1, RAB9B |
| 05321 | Inflammatory bowel disease (IBD) | 6 | **0.000805606** | **0.006835942** | GATA3, IL6, JUN, MAF, TGFB2, TLR5 |
| 05418 | Fluid shear stress and atherosclerosis | 8 | **0.000846748** | **0.006902283** | KLF2, DUSP1, HSP90AA1, JUN, MAP2K6, SUMO3, VEGFA, IL1R2 |
| 05142 | Chagas disease (American trypanosomiasis) | 7 | **0.000927847** | **0.007340907** | GNAO1, IL6, CXCL8, JUN, SERPINE1, CCL5, TGFB2 |
| 04620 | Toll-like receptor signaling pathway | 7 | **0.001020912** | **0.007846439** | IL6, CXCL8, IRF7, JUN, MAP2K6, CCL5, TLR5 |
| 05120 | Epithelial cell signaling in Helicobacter pylori infection | 6 | **0.001101723** | **0.008232319** | HBEGF, ATP6V0D2, CXCL8, JUN, ATP6V0A4, CCL5 |
| 05130 | Pathogenic Escherichia coli infection | 9 | **0.001626658** | **0.011826246** | CLDN4, F2, MYH15, IL6, CXCL8, JUN, PAK3, TLR5, CLDN2 |
| 04218 | Cellular senescence | 8 | **0.001841339** | **0.013034742** | GADD45A, IGFBP3, IL6, CXCL8, GADD45B, SERPINE1, MAP2K6, TGFB2 |
| 04935 | Growth hormone synthesis, secretion and action | 7 | **0.001966723** | **0.013565346** | IGFBP3, JUNB, SHC3, MAP2K6, SOCS1, SOCS3, CREB5 |
| 04810 | Regulation of actin cytoskeleton | 9 | **0.002237177** | **0.014748801** | VAV3, F2, ITGB7, ITGB8, PAK3, PDGFRA, CXCR4, ITGA10, FGF19 |
| 04141 | Protein processing in endoplasmic reticulum | 8 | **0.002247959** | **0.014748801** | CRYAB, DDIT3, PPP1R15A, HSPA1A, HSPA1B, HSP90AA1, RNF5, DERL3 |
| 04614 | Renin-angiotensin system | 4 | **0.002403099** | **0.015391275** | AGT, ENPEP, ANPEP, KLK2 |
| 04012 | ErbB signaling pathway | 6 | **0.002475064** | **0.01548354** | HBEGF, EREG, JUN, AREG, PAK3, SHC3 |
| 05166 | Human T-cell leukemia virus 1 infection | 9 | **0.002638815** | **0.016132757** | EGR1, IL6, JUN, LTA, NFKB2, TGFB2, IL1R2, FOSL1, CREB5 |
| 04926 | Relaxin signaling pathway | 7 | **0.002892162** | **0.016912863** | GNAO1, INSL3, JUN, SHC3, RLN2, VEGFA, CREB5 |
| 04512 | ECM-receptor interaction | 6 | **0.002854568** | **0.016912863** | LAMB4, TNC, ITGB7, ITGB8, LAMA4, ITGA10 |
| 04360 | Axon guidance | 8 | **0.003568451** | **0.019998192** | PLXNC1, EPHA7, UNC5B, PAK3, WNT4, RGMA, TRPC6, CXCR4 |
| 04621 | NOD-like receptor signaling pathway | 8 | **0.003568451** | **0.019998192** | HSP90AA1, TMEM173, IL6, CXCL8, IRF7, JUN, TRPV2, CCL5 |
| 04978 | Mineral absorption | 5 | **0.003934541** | **0.021599825** | SLC26A9, MT1F, MT1X, SLC8A1, TF |
| 05414 | Dilated cardiomyopathy (DCM) | 6 | **0.004070395** | **0.021898725** | AGT, ITGB7, ITGB8, SLC8A1, TGFB2, ITGA10 |
| 04750 | Inflammatory mediator regulation of TRP channels | 6 | **0.004799868** | **0.025316952** | TRPV3, IL1RAP, NGF, TRPV2, MAP2K6, TRPA1 |
| 05146 | Amoebiasis | 6 | **0.005197398** | **0.02545275** | LAMB4, IL6, CXCL8, LAMA4, TGFB2, IL1R2 |
| 04623 | Cytosolic DNA-sensing pathway | 5 | **0.005193239** | **0.02545275** | POLR3G, TMEM173, IL6, IRF7, CCL5 |
| 00410 | beta-Alanine metabolism | 4 | **0.005204094** | **0.02545275** | GAD1, AOC2, CARNS1, AOC3 |
| 00512 | Mucin type O-glycan biosynthesis | 4 | **0.005204094** | **0.02545275** | GALNT5, GALNT15, C1GALT1C1, GALNT4 |
| 04514 | Cell adhesion molecules (CAMs) | 7 | **0.005329834** | **0.025602239** | CNTN1, CLDN4, ITGB7, ITGB8, CADM3, MADCAM1, CLDN2 |
| 04072 | Phospholipase D signaling pathway | 7 | **0.005499033** | **0.025842561** | LPAR6, AGT, F2, CXCL8, PDGFRA, SHC3, SYK |
| 05226 | Gastric cancer | 7 | **0.00567213** | **0.025842561** | GADD45A, GADD45B, SHC3, WNT4, TGFB2, WNT1, FGF19 |
| 04625 | C-type lectin receptor signaling pathway | 6 | **0.005617693** | **0.025842561** | IL6, IRF1, JUN, NFKB2, BCL3, SYK |
| 04927 | Cortisol synthesis and secretion | 5 | **0.00576414** | **0.025842561** | AGT, LDLR, PDE8B, CACNA1G, CREB5 |
| 04659 | Th17 cell differentiation | 6 | **0.006292285** | **0.027747945** | GATA3, HSP90AA1, IL1RAP, IL6, IRF4, JUN |
| 04934 | Cushing syndrome | 7 | **0.006795434** | **0.029015424** | AGT, LDLR, WNT4, WNT1, PDE8B, CACNA1G, CREB5 |
| 05160 | Hepatitis C | 7 | **0.006795434** | **0.029015424** | CLDN4, IFIT1, IRF7, LDLR, MX1, SOCS3, CLDN2 |
| 05211 | Renal cell carcinoma | 5 | **0.007028256** | **0.029454235** | EGLN3, JUN, PAK3, TGFB2, VEGFA |
| 05020 | Prion diseases | 4 | **0.007117194** | **0.029454235** | EGR1, HSPA1A, IL6, CCL5 |
| 00350 | Tyrosine metabolism | 4 | **0.007652088** | **0.031188054** | ADH6, DDC, AOC2, AOC3 |
| 05161 | Hepatitis B | 7 | **0.008300348** | **0.03332528** | IL6, CXCL8, IRF7, JUN, MAP2K6, TGFB2, CREB5 |
| 04022 | cGMP-PKG signaling pathway | 7 | **0.00951253** | **0.037630451** | ADORA1, ADRA2B, EDNRA, KCNMB4, SLC8A1, TRPC6, CREB5 |
| 04612 | Antigen processing and presentation | 5 | **0.010509972** | **0.040973657** | HSPA1A, HSPA1B, HSP90AA1, KLRC2, KLRC3 |

**
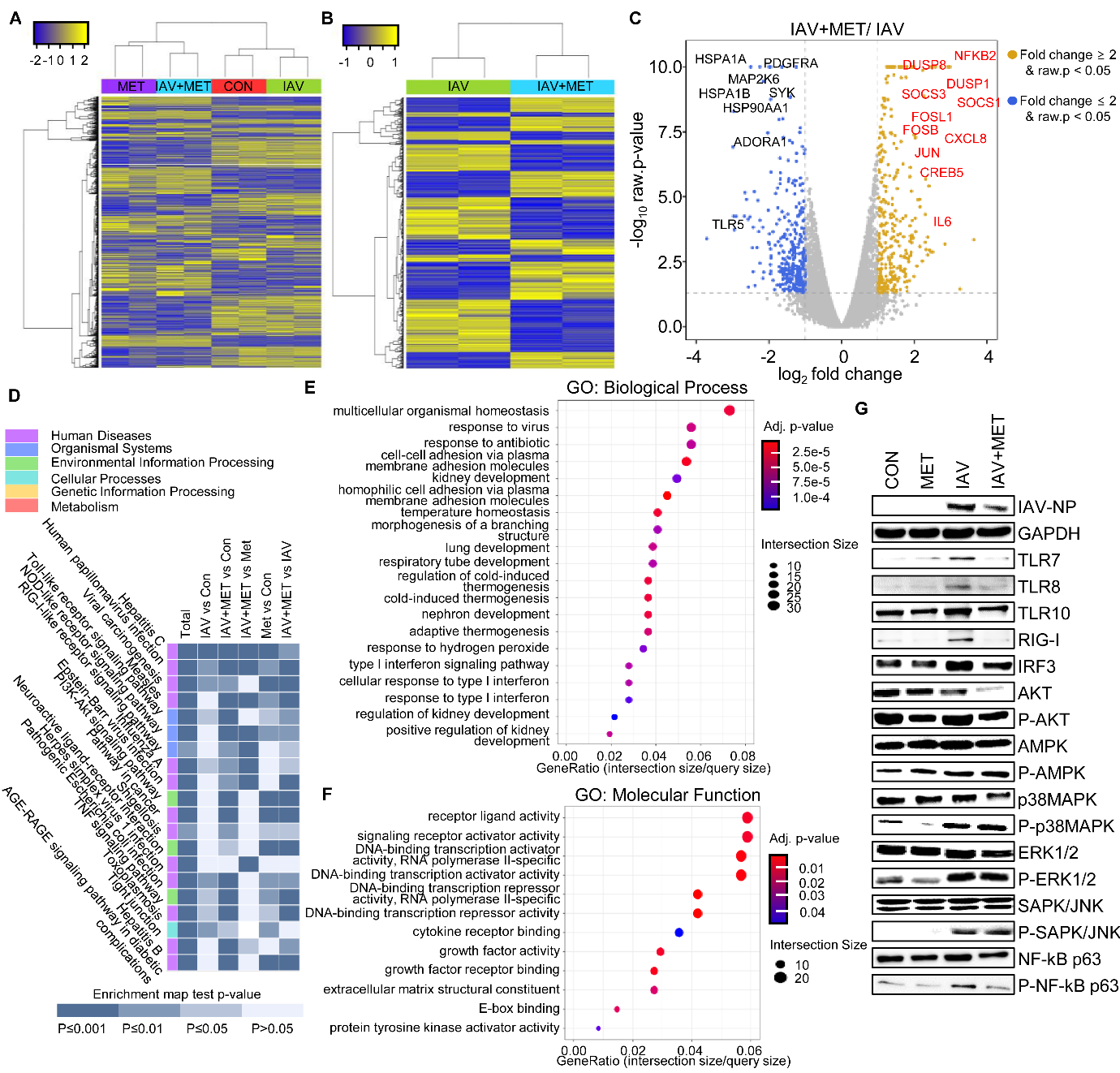
**

**Supplemental Figure 1. Biological pathways activated by influenza A virus infection are regulated by metformin.** The samples were divided into 4 groups: Control (CON), Metformin (MET), Influenza A virus infection (IAV), and metformin-treated infection (IAV+MET). After 3days of infection, mRNA was extracted from each group and RNA-seq was performed. The experiment was performed in two biological replicates. The fragment per kilobase of transcript per million mapped reads (FPKM) values ​​for each sample were summarized and differential expression analysis was performed. (**A** and **B)** Hierarchical clustering analysis for the list of significant differentially expressed genes (DEGs) and the degree of similarity by sample for each gene was determined. (**A**) The list of DEGs for the entire comparative combination and (**B**) the list of DEGs between the two groups were visualized by heatmaps and dendrograms. (**C)** The log2 fold change of the expression value and the p-value derived from the comparison of the mean between the two groups (IAV+MET and IAV) are represented by a Volcano plot. DEG, which was significantly increased more than two-fold, was indicated by a yellow dot. DEG, which was significantly decreased more than two-fold, was indicated by a blue dot. (**D)** Pathway enrichment analysis was performed on the significant DEGs based on the KEGG Pathway (http://www.kegg.jp/kegg/pathway.html). A heatmap is presented for the top 20 terms in enrichment test. The legend represents the enrichment p-value. (**E** and **F)** Gene ontology (GO) analysis was performed for significant DEGs in the IAV and Met-IAV groups. The results are presented as a dot plot. (**G)** Protein expression related to the pathway showing a significant difference in RNA-seq analysis was confirmed by Western blot. The phosphorylated form was indicated by P, such as P-AKT, P-p38MAPK. GAPDH is shown as the loading control. Refer to supplementary file for uncropped version of blots.


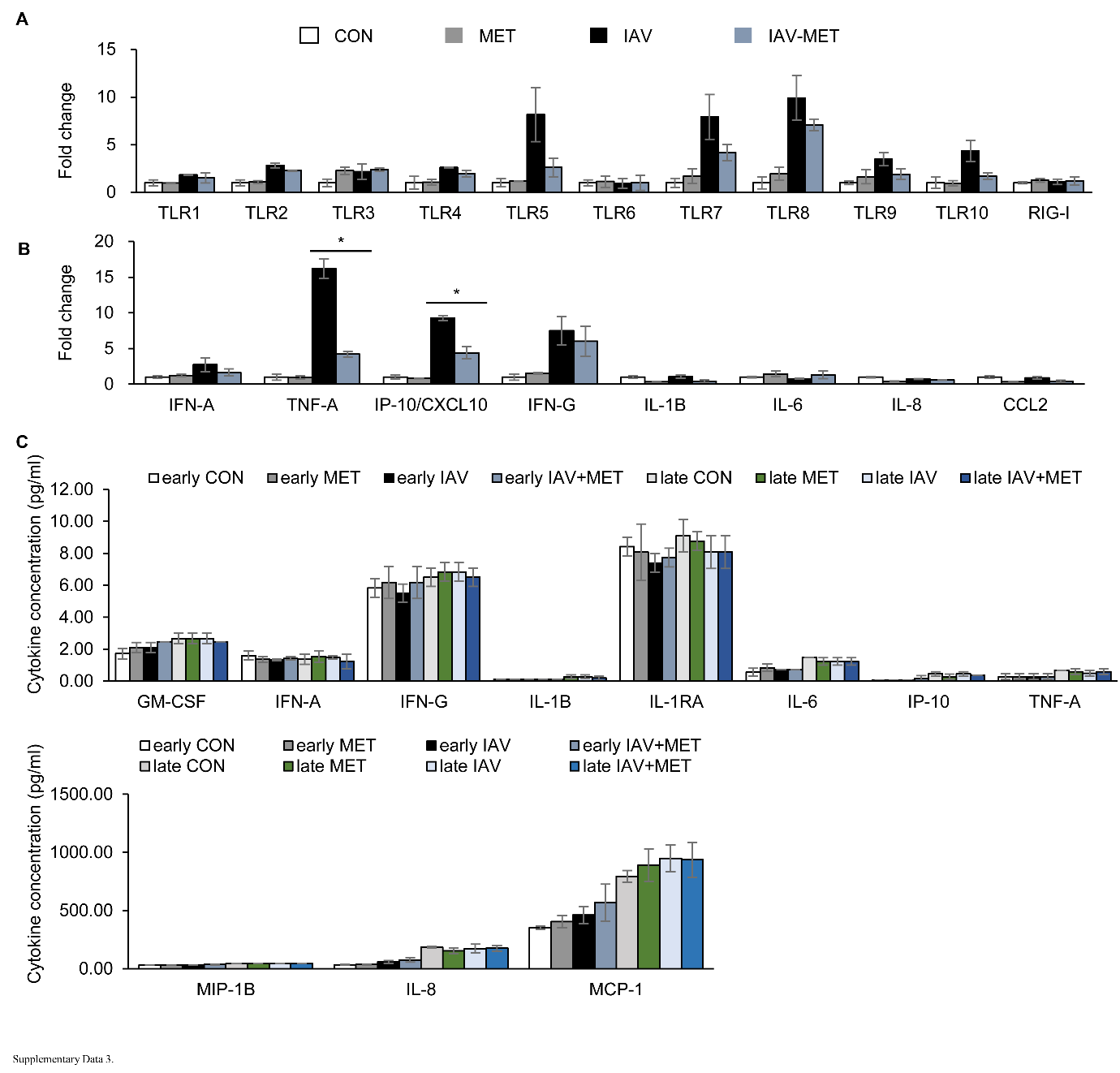


**Supplemental Figure 2. qRT-PCR and Luminex analysis in IAV-infected A549 cells.** Total RNA and supernatants were harvested at 48 hour after influenza A virus (IAV) infection. (**A**) RNA expression of Toll-like receptors and RIG-I were quantified using qRT-PCR. (**B**) Cytokine RNA level was measured by qRT-PCR. (**C**) The secretion of multiple cytokines in A549 cells cultured media was simultaneously measured using the Luminex system. Cytokines released at 8 (early) and 48 (late) hours after viral infection were measured. Data are shown as mean ± standard error of three independent experiments. Student’s t test was used statistically to compare group. **p* < 0.05.


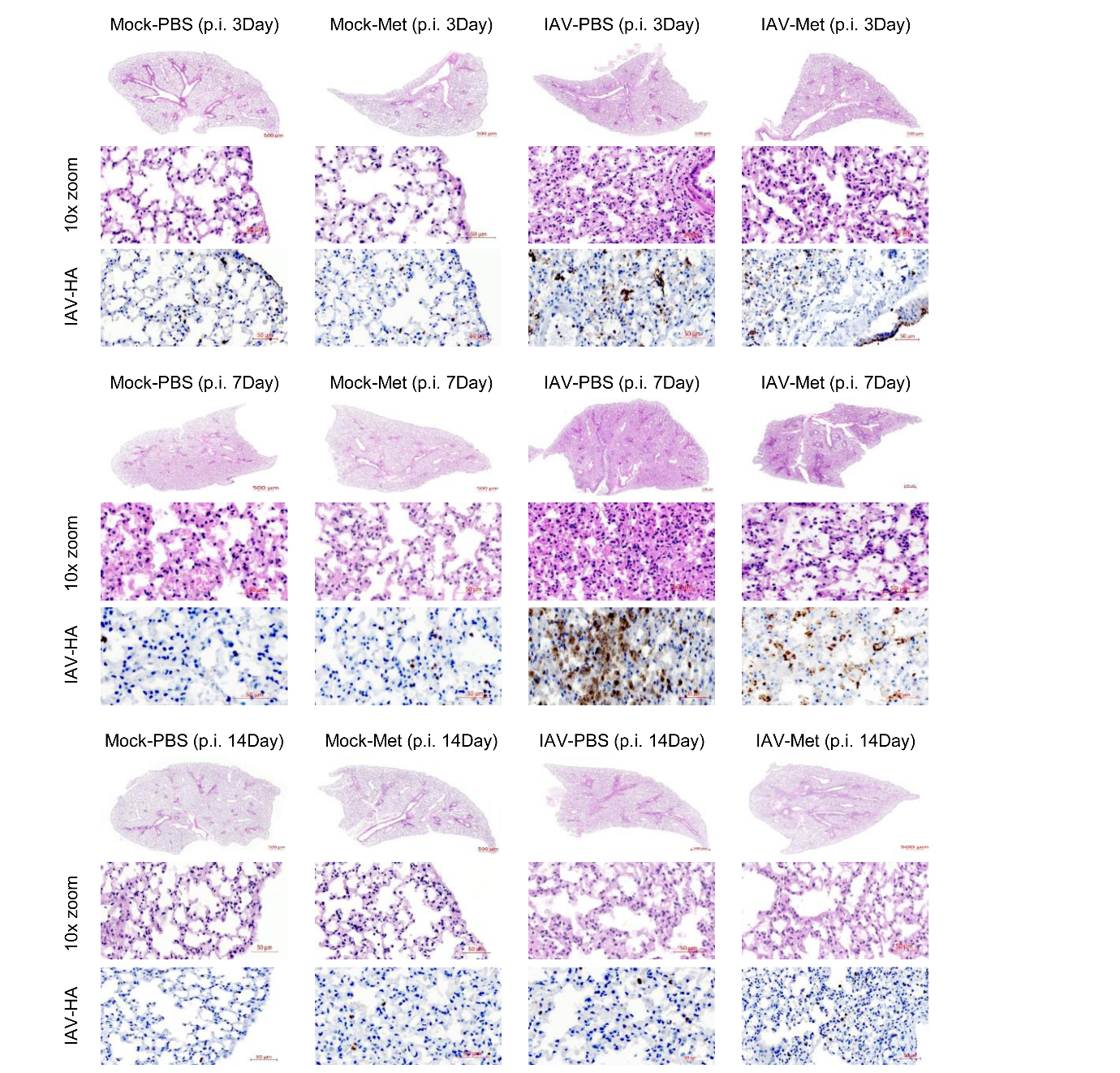


**Supplemental Figure 3. Virus replication and immune cell infiltration in the lungs of IAV-infected mice were reduced by metformin treatment.** High fat diet-induced atherosclerosis mice were intra-nasally infected with influenza A virus (Hong Kong/8/1968) and treated with or without 300 mg/kg/day metformin. (**A**) Mice lung sections were stained with anti-influenza hemagglutinin antibody (IAV-HA) or hematoxylin and eosin. Scale bars: 500 μm and 50 μm (10x zoomed-in image and IAV-HA stained image). (**B**) Mice aorta sections were stained with hematoxylin and eosin (H&E). Scale bars represent 100 μm.


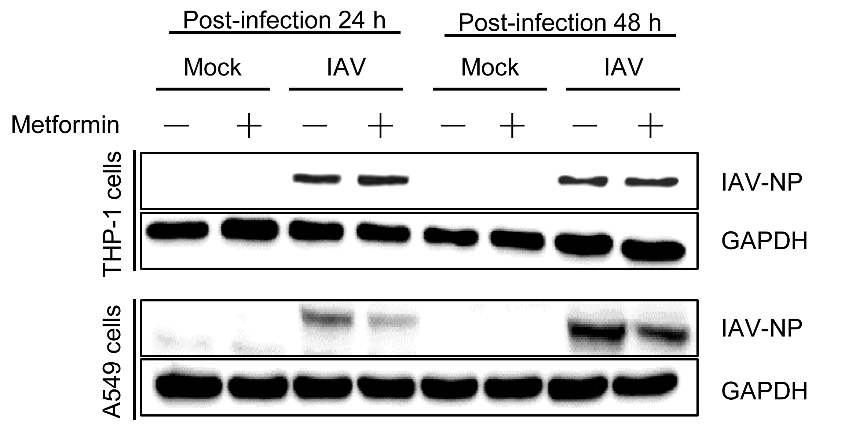


**Supplemental Figure 4. Differences in Influenza Virus Sensitivity by Cells.** Virus infection was indicated by NP protein detection (IAV-NP). GAPDH was shown as loading control. Refer to supplementary file for uncropped version of blots.
